# Prognostic Associations of Concomitant Antibiotic Use in Patients with Advanced NSCLC Treated with Atezolizumab: Sensitivity Analysis of a Pooled Investigation of Five Randomised Control Trials

**DOI:** 10.3390/biomedicines11020528

**Published:** 2023-02-11

**Authors:** Arkady T Manning-Bennett, Julie Cervesi, Pierre-Alain Bandinelli, Michael J Sorich, Ashley M Hopkins

**Affiliations:** 1 College of Medicine and Public Health, Flinders University, Adelaide 5042, Australia; 2 Da Volterra, 56048 Paris, France

**Keywords:** non-small cell lung cancer, atezolizumab, antibiotics

## Abstract

Background: Immune checkpoint inhibitors (ICIs) have been a significant milestone for the treatment of advanced non-small cell lung cancer (NSCLC). However, the efficacy of ICIs can vary substantially between patients, with disparities in treatment outcomes being potentially driving by changes in the microbiome. Antibiotics can cause dysbiosis and are hypothesised to impact the efficacy of ICIs Methods: Data were pooled from five randomised clinical control trials, IMpower130, IMpower131, IMpower150, OAK, and POPLAR, assessing atezolizumab in advanced NSCLC. Cox proportional hazard models were used to determine whether antibiotic use within 6-weeks before and after randomisation was associated with progression-free survival (PFS) and overall survival (OS) outcomes, with data further stratified by programmed death ligand-1 (PD-L1) status. Results: Antibiotic use was significantly associated with worsened PFS (hazard ratio (HR) = 1.19 [1.08–1.30], *p* ≤ 0.001) and OS (HR = 1.27 [1.13–1.42], *p* ≤ 0.001) in patients treated with atezolizumab and those not treated with atezolizumab (PFS, HR = 1.21 [1.08–1.36] *p* < 0.001, OS, HR = 1.33 [1.16–1.51] *p* < 0.001). These associations were relatively consistent in both PD-L1 positive and PD-L1 negative. Conclusions: Antibiotic use within a ±6-week window was significantly associated with worse PFS and OS.

## 1. Introduction

Immune checkpoint inhibitors (ICIs) have been a significant advancement in the treatment arsenal for advanced non-small cell lung cancer (NSCLC) [1,2,3,4]. However, the efficacy of ICIs can vary among patients, and hypotheses are that this may be partly driven by differences in gut microbiota health between patients [5]. Antibiotics cause gut dysbiosis and thus hypotheses are that antibiotics may decrease the effectiveness of ICIs—however, evidence of casual impacts on ICI efficacies remains debated [6,7]. In part, this is because of complexities around the ethicality of conducting a prospective study assessing if antibiotics worsen the efficacy of ICIs in patients without infections, and in patients with infections antibiotics cannot be withheld. Consequently, much research has used retrospective data, from which making casual inferences has limitations—for example, corticosteroid use, programmed death ligand-1 (PD-L1) expression, and the time-window of antibiotic use may cause confounding. Meanwhile, studies evaluating associations of antibiotic use and objective response in advanced NSCLC patients treated with ICIs, as well as outcomes in patients receiving treatments excluding ICIs, have been limited.

Our team recently published a pooled analysis of randomised control trials (RCTs) IMpower130 (NCT02367781), IMpower131 (NCT02367794), IMpower150 (NCT02366143), OAK (NCT02008227), and POPLAR (NCT01903993)—RCTs evaluating the efficacy of the ICI atezolizumab in patients with advanced NSCLC. Given the hypothesis that non-cancer medications may disrupt the gut microbiome and impact ICI treatment, we previously evaluated the efficacy of atezolizumab in the context of antibiotic and proton-pump inhibitor use [8,9,10,11]. Within our previous study (1), antibiotic use within 30 days prior to atezolizumab initiation and antibiotic use in the 30 days post treatment initiation were compared against patients who did not use any antibiotics within that time period—in an analysis adjusted for age, sex, race, Eastern Cooperative Oncology Group (ECOG) performance status, smoking status, histology, presence of liver metastases, and PD-L1 expression, it was identified that antibiotic use in the 30 days post treatment initiation was strongly associated with worsened overall survival and progression free survival. It was also observed that the frequency of post-atezolizumab-initiation antibiotic use was 215% greater than the frequency of antibiotic use within the 30 days prior to treatment initiation (*n* = 612 versus 194, respectively). Our team have received considerable comments to present analyses which are adjusted for corticosteroid use, analyse alternate time-windows of antibiotic use, and evaluate the potential impacts according to PD-L1 expression [12,13,14]. We aimed to present additional data adjusted for corticosteroid use, subset by PD-L1 expression (herein defined as PD-L1 positive (tumour expression ≥ 1%) and PD-L1 negative (tumour expression of <1%)), an antibiotic use time window of ±42 days from treatment randomisation, and objective response.

## 2. Materials and Methods

The analyses followed methodologies previously described [8]. Individual participant data (IPD) were pooled from five randomised control trials analysing atezolizumab against chemotherapy in metastatic NSCLC patients. The data were analysed in the format of an IPD level meta-analysis. The pooled trials were: IMpower130 (NCT02367781), IMpower131 (NCT02367794), IMpower150 (NCT02366143), OAK (NCT02008227), and POPLAR (NCT01903993). Data were collated on study participants who received at least one dose of an antibiotic within a window of 42 days prior to randomisation to 42 days after randomisation. Cox proportional hazard analyses were used to determine whether this antibiotic use was associated with differences in progression free survival (PFS) or overall survival (OS) [15]. Logistic regression was used to evaluated associations with objective response. Subgroup analyses of the prognostic association of antibiotic use according to PD-L1 expression were conducted. Positive PD-L1 status was defined as a PD-L1 tumour expression of ≥1%, and negative PD-L1 status as a tumour expression of <1%. Models were adjusted for age, weight, sex, ethnicity, smoking status (current, previous, never), presence of tumour metastases, concomitant proton pump inhibitor use (designated as use within a ±42 day [i.e., ±6 week] window from randomisation), and concomitant corticosteroid use (designated as being prescribed two or more doses within a ±42 day window from randomisation), with models stratified by baseline Eastern Cooperative Oncology Group performance status (ECOG-PS) score, clinical trial, and intent to treat treatment arm. Results were presented as hazard ratios (HR) or odds ratios (OR) with 95% confidence interval [95%-CI]. All analyses were conducted within the R software.

This research is based on data from Roche that has been made available through Vivli, Inc. Vivli has not contributed to or approved, and is not in any way responsible for, the contents of this publication. All clinical trials used in analysis were undertaken in accordance with the Declaration of Helsinki. Informed consent was obtained from all subjects involved in the clinical trials. Secondary analysis of anonymised clinical trial data was confirmed negligible risk research by the Southern Adelaide Local Health Network, Office for Research and Ethics, and was exempt from review.

## 3. Results

The pooled cohort included 4459 study participants; 2724 treated with atezolizumab ± chemotherapy and 1735 receiving a treatment not including atezolizumab. Of the 2724 participants who received atezolizumab, 846 (31%) used antibiotics within ±42 days of atezolizumab initiation (Supplementary Table S1). Of the 1735 receiving treatment with chemotherapy (±bevacizumab), 525 (30%) used antibiotics within ±42 days of treatment initiation. The most commonly prescribed antibiotics were quinolone-based (Supplementary Table S2), with most antibiotics prescribed after randomisation (Supplementary Table S3).

**Figure 1:**
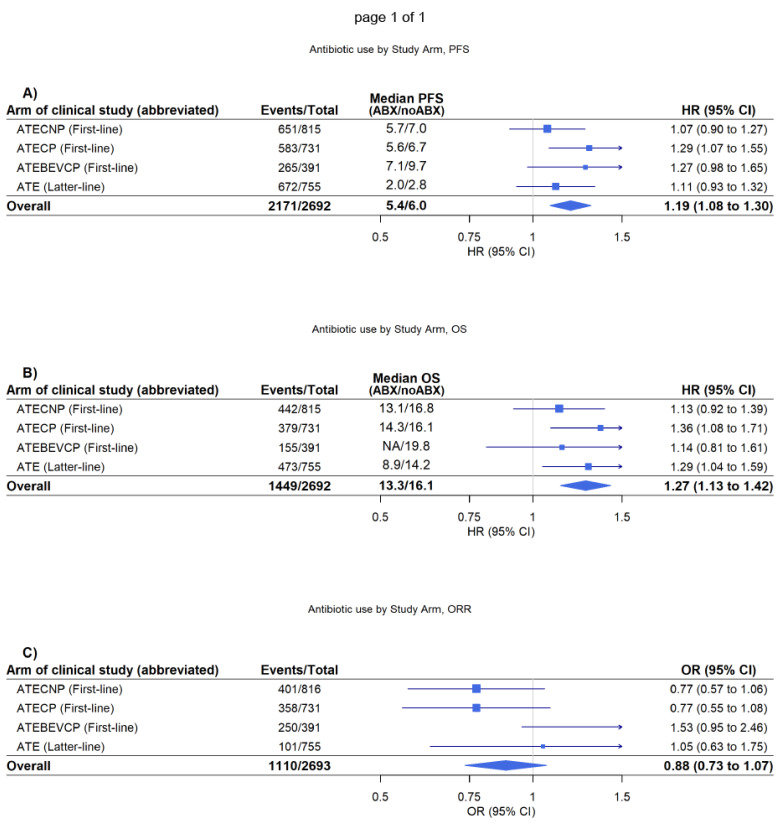
ATECNP (atezolizumab with carboplatin and nab-paclitaxel), ATECP (atezolizumab with carboplatin and paclitaxel), ATEBEVCP (atezolizumab with bevacizumab, carboplatin, and paclitaxel), and ATE (atezolizumab monotherapy). Forest plot of the adjusted association between antibiotic use and prognosis by study arm in the cohort randomised therapies with atezolizumab. Median time to progression-free survival (PFS) and overall survival (OS) are unadjusted. (**A**) Forest plot of association of antibiotics with PFS. (**B**) Forest plot of association of antibiotics with OS. (**C**) Forest plot of association of antibiotics with objective response rate (ORR).

In analyses adjusted for age, weight, sex, ethnicity, smoking status, presence of tumour metastases, proton pump inhibitor use, and corticosteroid use, and stratified by ECOG performance status, trial and arm, antibiotic use (within ±42 days of randomisation) was significantly associated with worsened OS and PFS in patients randomised to treatment regimens including atezolizumab monotherapy and atezolizumab in combination with chemotherapy (PFS, HR = 1.19 [1.08–1.30], *p* ≤ 0.001, OS, HR = 1.27 [1.13–1.42], *p* ≤ 0.001) (Figure 1). These associations were relatively consistent in both the PD-L1 positive and PD-L1 negative cohorts (Supplementary Figures S1 and S2)—albeit the magnitude of the worsening appears higher in the PD-L1 positive cohort (e.g., the HR point estimate for OS was 1.52 for the PD-L1 positive cohort versus 1.18 for the PD-L1 negative cohort). Antibiotic use was not associated with a difference in objective response rate (OR = 0.88 [0.73–1.07], *p* = 0.196) (Figure 1).

**Figure 2:**
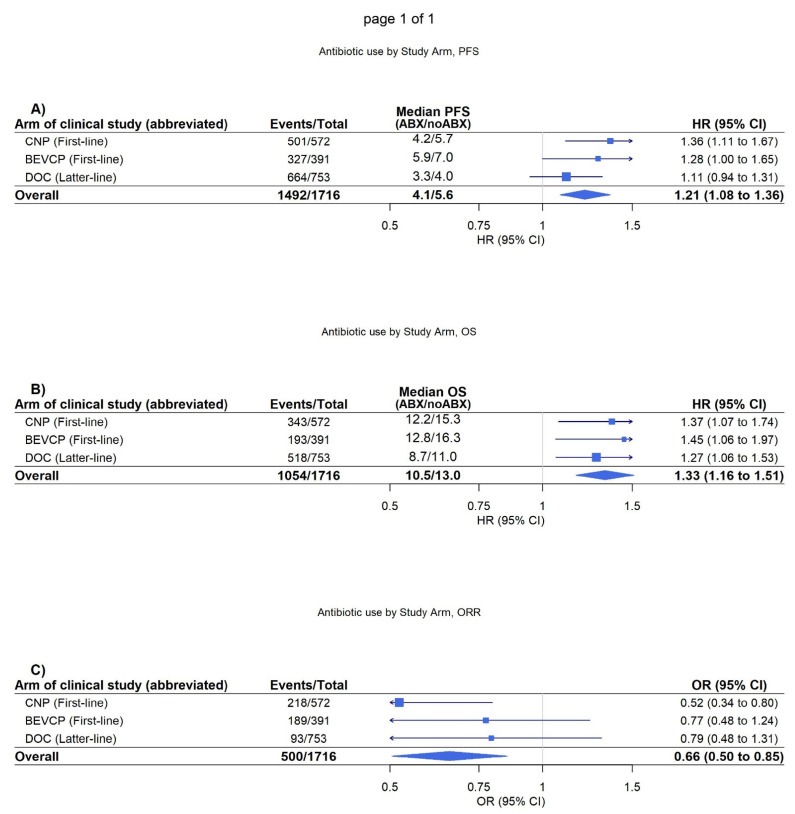
CNP (carboplatin and nab-paclitaxel), BEVCP (bevacizumab, carboplatin, and paclitaxel), and DOC (docetaxel). Forest plot of the adjusted association between antibiotic use and prognosis by study arm in the cohort randomised therapies without atezolizumab. Median time to progression-free survival (PFS) and overall survival (OS) are unadjusted. (**A**) Forest plot of association of antibiotics with PFS. (**B**) Forest plot of association of antibiotics with OS. (**C**) Forest plot of association of antibiotics with objective response rate (ORR).

It was further identified on adjusted analysis that antibiotic use was significantly associated with worsened OS, PFS, and objective response in patients randomised to treatment regimens not including atezolizumab (PFS, HR = 1.21 [1.08–1.36] *p* < 0.001, OS, HR = 1.33 [1.16–1.51] *p* < 0.001, objective response, OR = 0.66 [0.51–0.85], *p* = 0.002) (Figure 2). These associations were again demonstrated as consistent in both the PD-L1 positive and PD-L1 negative cohorts (Supplementary Figures S3 and S4).

## 4. Discussion

In a pooled cohort of 4459 study participants (2724 receiving atezolizumab and 1735 not receiving atezolizumab), antibiotic use within a ± 42 day time-window from randomization was identified as significantly associated with poorer PFS and OS in both atezolizumab and chemotherapy treated patients. These results are like those from our previous analysis in NSCLC [8], but they importantly show that extending the antibiotic use window from 30 to 42 days (i.e., 6 weeks) and adjusting for corticosteroid use did not significantly change the results.

To the best of our knowledge, this study presents the largest exploratory analysis of RCT data investigating the association between antibiotic use within a ±42 day time-window with survival outcomes in patients treated with both atezolizumab ± chemotherapy and chemotherapy alone. The analysis was undertaken due to comments to present analyses which are adjusted for corticosteroid use, analyses with an extended time-window of antibiotic use and to evaluate the potential impacts according to PD-L1 expression [12,13,14]. The results of the present study are particularly reassuring for current clinical practice as they reaffirm that ICIs are still a beneficial treatment in NSCLC despite concomitant or previous antibiotic use within a large time window. Thus, while antibiotic use is a significant negative prognostic marker for individuals treated with atezolizumab ± chemotherapy, the results of the study also demonstrate this to be true of the observed association for individuals treated with chemotherapy alone. As such, more research on how antibiotics interact with ICI therapy is needed before there are any changes to clinical practice.

A limitation of this study was a lack of ability to evaluate the dose, duration, or compliance to antibiotic therapy. The clinical trial origins of the data may also limit generalizability of findings to real world populations. Nonetheless, randomization provides validity to between treatment comparisons, the study is the largest to date with the 6-week time-window, and the clinical trial nature ensures the data are highly regulated and of high-quality. Furthermore, we were unable to determine if the aforementioned associations varied in patients with high PD-L1 (≥50%) compared with low (≥1–49%) or negative PD-L1 tumour expression, due to limitations on sample size in the PD-L1 high groups. Future research should focus on determining if the observed negative associations of antibiotics with outcomes in patients treated with both atezolizumab ± chemotherapy or chemotherapy alone are driven by the antibiotics themselves or the indications they’re prescribed for—this is particularly pertinent given the potential for confounding by indication, as patients who are generally more ill are more likely to be prescribed antibiotics. If the former, then the negative impacts of antibiotics on the gut microbiome may extend to therapies beyond ICIs. Meanwhile, investigating the impact of specific antibiotic classes—which may be associated with different gut dysbiosis effects—will also be of interest.

In conclusion, the present study identified that antibiotic use within a ±42 day (i.e., 6 week) window was significantly associated with worse PFS and OS in patients treated with both atezolizumab ± chemotherapy or chemotherapy alone. These associations were relatively consistent in both PD-L1 positive and PD-L1 negative patients, and were observed to be independent of concomitant corticosteroid use (as adjusted for the use of two or more doses within a ±42-day window from randomisation).

## Data Availability

All data utilised in analysis are available through Vivli.

## Supplementary Materials

The following supporting information can be downloaded at: https://www.mdpi.com/article/10.3390/biomedicines11020528/s1, Figure S1: Antibiotic use by study arm, PDL-1 positive atezolizumab patients, Figure S2: Antibiotic use by study arm, PDL-1 negative atezolizumab patients, Figure S3: Antibiotic use by study arm, PDL-1 positive non-atezolizumab patients, Figure S4: Antibiotic use by study arm, PDL-1 negative non-atezolizumab patients; Table S1: Demographics, Table S2: Antibiotic Class, Table S3: Antibiotic Use.

## References

[1] Darvin P., Toor S.M., Sasidharan Nair V., Elkord E. (2018). Immune checkpoint inhibitors: Recent progress and potential biomarkers. Exp. Mol. Med..

[2] Reck M., Rodríguez-Abreu D., Robinson A.G., Hui R., Csőszi T., Fülöp A., Gottfried M., Peled N., Tafreshi A., Cuffe S. (2016). Pembrolizumab versus Chemotherapy for PD-L1–Positive Non–Small-Cell Lung Cancer. N. Engl. J. Med..

[3] Socinski M.A., Jotte R.M., Cappuzzo F., Orlandi F., Stroyakovskiy D., Nogami N., Rodríguez-Abreu D., Moro-Sibilot D., Thomas C.A., Barlesi F. (2018). Atezolizumab for First-Line Treatment of Metastatic Nonsquamous NSCLC. N. Engl. J. Med..

[4] Hellmann M.D., Paz-Ares L., Bernabe Caro R., Zurawski B., Kim S.-W., Carcereny Costa E., Park K., Alexandru A., Lupinacci L., de la Mora Jimenez E. (2019). Nivolumab plus Ipilimumab in Advanced Non–Small-Cell Lung Cancer. N. Engl. J. Med..

[5] Routy B., Le Chatelier E., Derosa L., Duong C.P.M., Alou M.T., Daillère R., Fluckiger A., Messaoudene M., Rauber C., Roberti M.P. (2018). Gut microbiome influences efficacy of PD-1-based immunotherapy against epithelial tumors. Science.

[6] Wilson B.E., Routy B., Nagrial A., Chin V.T. (2020). The effect of antibiotics on clinical outcomes in immune-checkpoint blockade: A systematic review and meta-analysis of observational studies. Cancer Immunol. Immunother..

[7] Yu Y., Zheng P., Gao L., Li H., Tao P., Wang D., Ding F., Shi Q., Chen H. (2021). Effects of Antibiotic Use on Outcomes in Cancer Patients Treated Using Immune Checkpoint Inhibitors: A Systematic Review and Meta-Analysis. J. Immunother..

[8] Hopkins A.M., Badaoui S., Kichenadasse G., Karapetis C.S., McKinnon R.A., Rowland A., Sorich M.J. (2022). Efficacy of Atezolizumab in Patients With Advanced NSCLC Receiving Concomitant Antibiotic or Proton Pump Inhibitor Treatment: Pooled Analysis of Five Randomized Control Trials. J. Thorac. Oncol..

[9] Hopkins A.M., Kichenadasse G., Karapetis C.S., Rowland A., Sorich M.J. (2020). Concomitant Proton Pump Inhibitor Use and Survival in Urothelial Carcinoma Treated with Atezolizumab. Clin. Cancer Res..

[10] Hopkins A.M., Kichenadasse G., Karapetis C.S., Rowland A., Sorich M.J. (2020). Concomitant Antibiotic Use and Survival in Urothelial Carcinoma Treated with Atezolizumab. Eur. Urol..

[11] Hopkins A.M., Kichenadasse G., McKinnon R.A., Abuhelwa A.Y., Logan J.M., Badaoui S., Karapetis C.S., Rowland A., Sorich M.J. (2022). Efficacy of first-line atezolizumab combination therapy in patients with non-small cell lung cancer receiving proton pump inhibitors: Post hoc analysis of IMpower150. Br. J. Cancer.

[12] Cortellini A., Facchinetti F., Derosa L., Pinato D.J. (2022). Antibiotic Exposure and Immune Checkpoint Inhibitors in Patients With NSCLC: The Backbone Matters. J. Thorac. Oncol..

[13] Hopkins A.M., Sorich M.J. (2022). Response to Mazzaschi and Buti. J. Thorac. Oncol..

[14] Mazzaschi G., Buti S. (2022). What Is the Real Impact of Concomitant Antibiotics or Proton Pump Inhibitors on Efficacy of Atezolizumab-Based Regimens in Patients With NSCLC?. J Thorac Oncol.

[15] Cox D.R. (1972). Regression Models and Life-Tables. J. R. Stat. Society. Ser. B (Methodol.).

