# Supplementary material for: Prognostic Associations of Concomitant Antibiotic Use in Patients with Advanced NSCLC Treated with Atezolizumab: Sensitivity Analysis of a Pooled Investigation of Five Randomised Control Trials
Source: Biomedicines. 2023 Feb 11;11(2):528. doi: 10.3390/biomedicines11020528 (PMC9953420; doi:10.3390/biomedicines11020528)
Supplement: Supplementary file 1 [file biomedicines-11-00528-s001.zip › biomedicines-2132167-supplementary.pdf]

Table S1 Demographics, stratified by whether a patient was classified as being on antibiotics within the 6 week window

Summary of patient characteristics by antibiotics, +/- 6 weeks				
	**Total No. 4,459**	**No No. 3,088**	**Yes No. 1,371**	**P-value**
Study				0.49
IMPOWER130	724 (16%)	492 (16%)	232 (17%)	
IMPOWER131	1,021 (23%)	722 (23%)	299 (22%)	
IMPOWER150	1,202 (27%)	826 (27%)	376 (27%)	
OAK	1,225 (27%)	840 (27%)	385 (28%)	
POPLAR	287 (6%)	208 (7%)	79 (6%)	
Arm of clinical study (abbreviated)				0.002
ATE	757 (17%)	553 (18%)	204 (15%)	
ATEBEVCP	400 (9%)	265 (9%)	135 (10%)	
ATECNP	827 (19%)	566 (18%)	261 (19%)	
ATECP	740 (17%)	494 (16%)	246 (18%)	
BEVCP	400 (9%)	292 (9%)	108 (8%)	
CNP	580 (13%)	423 (14%)	157 (11%)	
DOC	755 (17%)	495 (16%)	260 (19%)	
ATEFL	2,724 (61%)	1,878 (61%)	846 (62%)	0.60
Age (years)	64 (57 - 70)	63 (57 - 69)	65 (58 - 71)	0.002
Sex				0.25
Male	2,896 (65%)	2,023 (66%)	873 (64%)	
Female	1,563 (35%)	1,065 (34%)	498 (36%)	
ECOG PS				0.009
0	1,695 (38%)	1,213 (39%)	482 (35%)	
1+	2,752 (62%)	1,866 (60%)	886 (65%)	
Missing	12 (<1%)	9 (<1%)	3 (<1%)	
Weight (kg)				0.16
Median (IQR)	71 (61 - 83)	72 (61 - 83)	71 (60 - 83)	
Missing	38 (1%)	38 (1%)	0 (0%)	
Liver tumor site at baseline	748 (17%)	509 (16%)	239 (17%)	0.46
Smoking history				< 0.001
Never	669 (15%)	473 (15%)	196 (14%)	
Previous	2,815 (63%)	1,880 (61%)	935 (68%)	
Current	974 (22%)	734 (24%)	240 (18%)	
Missing	1 (<1%)	1 (<1%)	0 (0%)	
Histology				0.87
Non-squamous	3,021 (68%)	2,095 (68%)	926 (68%)	
Squamous	1,438 (32%)	993 (32%)	445 (32%)	
PDL1_Grouping				0.082
Negative	3,026 (68%)	2,121 (69%)	905 (66%)	
Positive	1,423 (32%)	960 (31%)	463 (34%)	
Missing	10 (<1%)	7 (<1%)	3 (<1%)	
PPI - Prior concomitant or up to 42 days	1,352 (30%)	805 (26%)	547 (40%)	< 0.001
Corticosteroids - PRE or POST 42 days	1,933 (43%)	1,193 (39%)	740 (54%)	< 0.001

Summary of patient characteristics by antibiotics, +/- 6 weeks				
	**Total**<br>**No. 4,459**	**No**<br>**No. 3,088**	**Yes**<br>**No. 1,371**	**P-value**

Data are median (IQR) or number of patients (%). P values per Chi-Square test for categorical data and Kruskal-Wallis test for continuous data.

Table S2 Breakdown of antibiotic class across the total number of antibiotic prescriptions within the 6 week window

Summary of patient characteristics	
	**Total No. 2,793**
Medication class	
QUINOLONE ANTIBIOTICS	768 (27%)
PENICILLINS	688 (25%)
CEPHALOSPORIN ANTIBIOTICS	411 (15%)
MACROLIDE ANTIBIOTICS	160 (6%)
GLYCOPEPTIDE ANTIBIOTICS	145 (5%)
MISCELLANEOUS ANTIMICROBIALS	141 (5%)
CARBAPENEM ANTIBIOTICS	106 (4%)
TETRACYCLINES	68 (2%)
AMINOGLYCOSIDE ANTIMICROBIALS	64 (2%)
[13 other]	242 (9%)

Data are median (IQR) or number of patients (%).

Table S3 Antibiotic use, stratified by whether the patient was classified as having antibiotics before or after treatment

Summary of patient characteristics	
	**Total No. 1,371**
Antibiotics - Concomitant or 42 days PRE treatment initiation	288 (21%)
Antibiotics - 42 days POST	1,195 (87%)

Data are median (IQR) or number of patients (%).

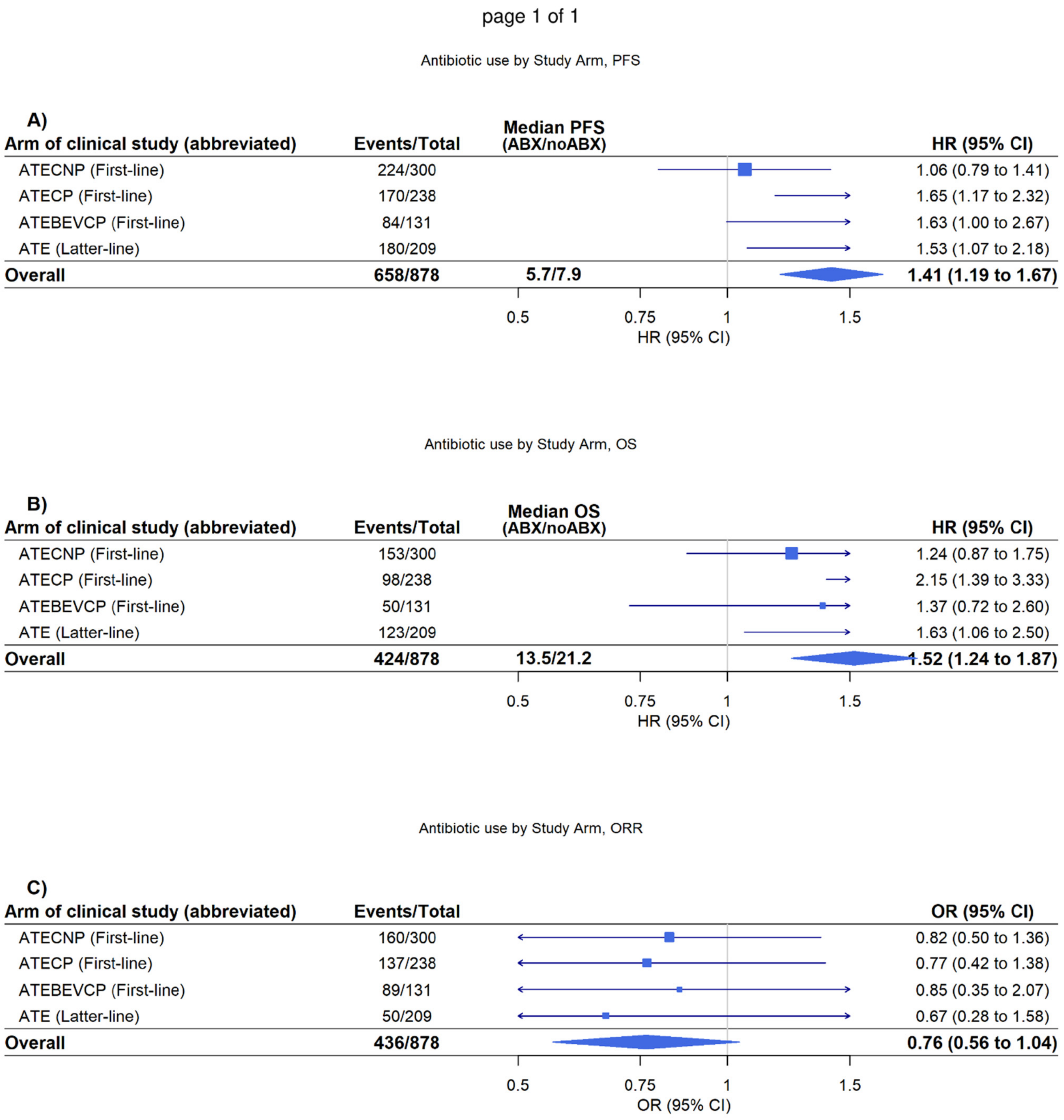

Figure S1: ATECNP (atezolizumab with carboplatin and nab-paclitaxel), ATECP (atezolizumab with carboplatin and paclitaxel), ATEBEVCP (atezolizumab with bevacizumab, carboplatin and paclitaxel), ATE (atezolizumab monotherapy).
Forest plot of the adjusted association between antibiotic use and prognosis by study arm in the **PDL-1 positive** cohort randomised therapies **with** atezolizumab. Median time to progression-free survival (PFS) and overall survival (OS) are unadjusted.
A) Forest plot of association of antibiotics with PFS.
B) Forest plot of association of antibiotics with OS.
C) Forest plot of association of antibiotics with objective response rate (ORR).

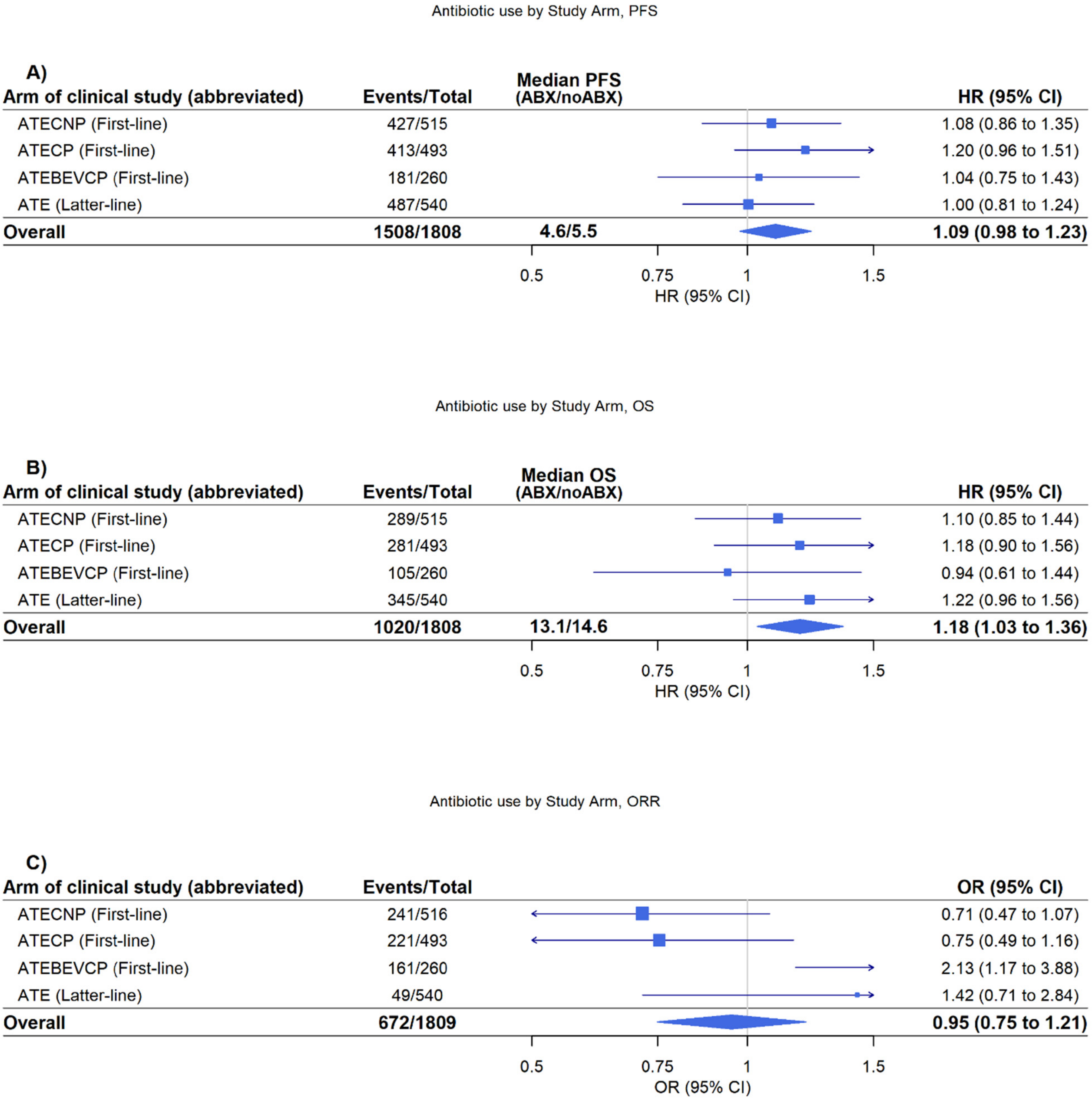

Figure S2: ATECNP (atezolizumab with carboplatin and nab-paclitaxel), ATECP (atezolizumab with carboplatin and paclitaxel), ATEBEVCP (atezolizumab with bevacizumab, carboplatin and paclitaxel), ATE (atezolizumab monotherapy).

Forest plot of the adjusted association between antibiotic use and prognosis by study arm in the **PDL-1 negative** cohort randomised therapies **with** atezolizumab. Median time to progression-free survival (PFS) and overall survival (OS) are unadjusted.

A) Forest plot of association of antibiotics with PFS.

B) Forest plot of association of antibiotics with OS.

C) Forest plot of association of antibiotics with objective response rate (ORR).

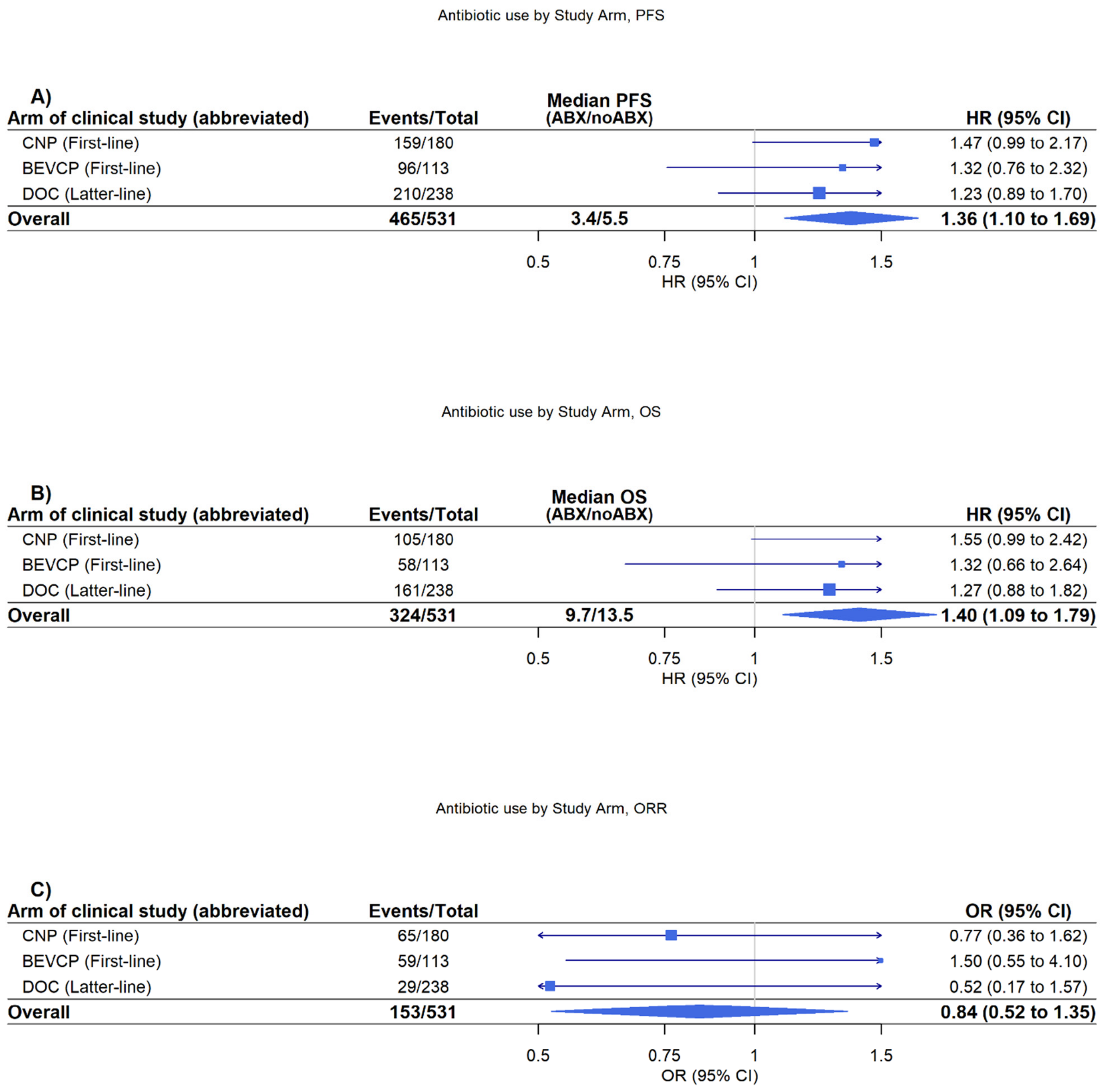

Figure S3: Forest plot of the adjusted association between antibiotic use and prognosis by study arm in the **PDL-1 positive** cohort randomised therapies **without** atezolizumab. Median time to progression-free survival (PFS) and overall survival (OS) are unadjusted.

A) Forest plot of association of antibiotics with PFS.

B) Forest plot of association of antibiotics with OS.

C) Forest plot of association of antibiotics with objective response rate (ORR).

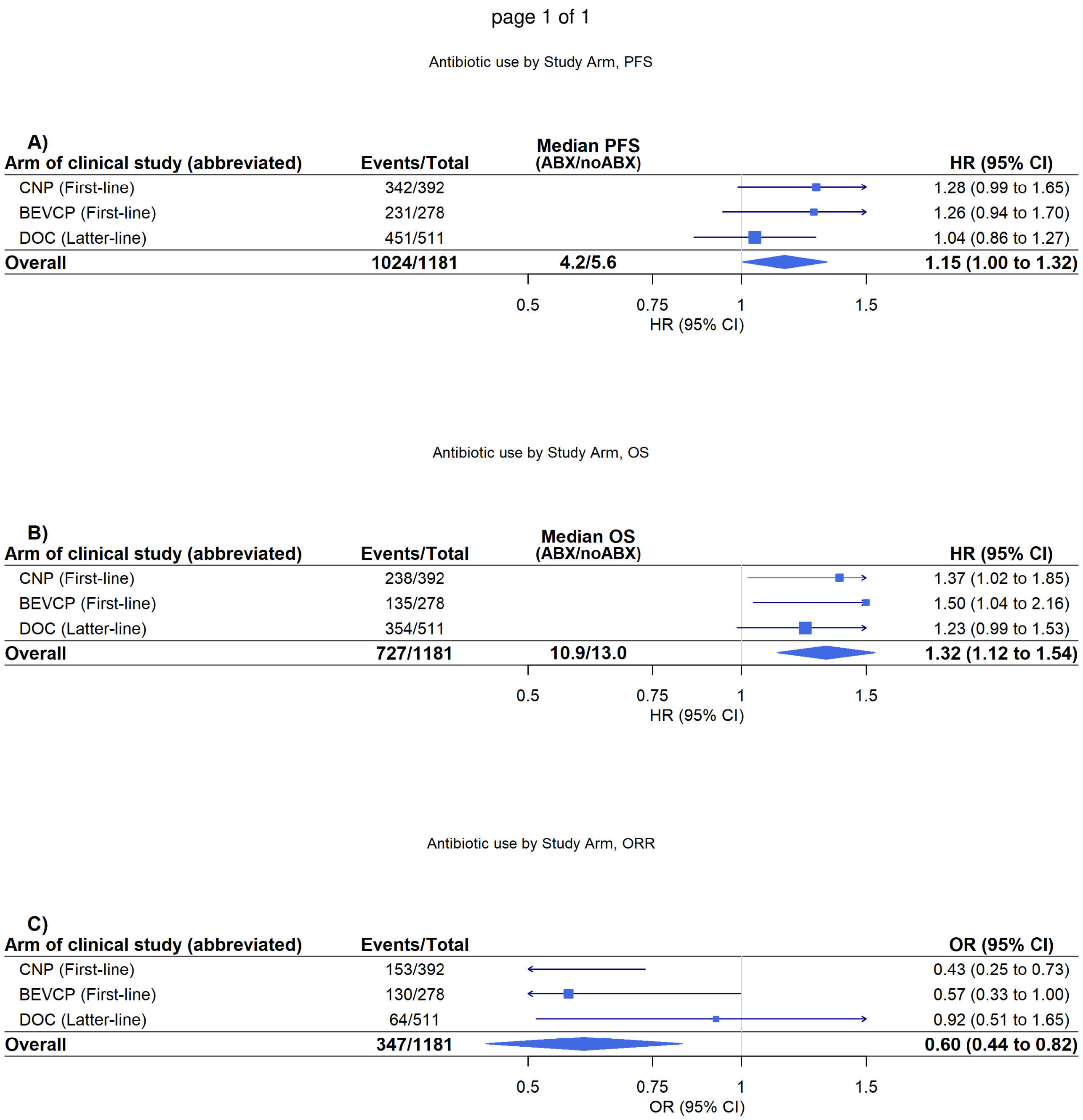

Figure S4: Forest plot of the adjusted association between antibiotic use and prognosis by study arm in the **PDL-1 negative** cohort randomised therapies **without** atezolizumab. Median time to progression-free survival (PFS) and overall survival (OS) are unadjusted.

A) Forest plot of association of antibiotics with PFS.

B) Forest plot of association of antibiotics with OS.

C) Forest plot of association of antibiotics with objective response rate (ORR)
